# Metabolic complete response with vinflunine as second-line therapy in a kidney-transplanted patient with advanced urothelial carcinoma: a case report

**DOI:** 10.1186/s12885-016-2666-6

**Published:** 2016-08-12

**Authors:** Paola Bordi, Marcello Tiseo, Giorgio Baldari, Sebastiano Buti

**Affiliations:** 1 Medical Oncology Unit, University Hospital of Parma, Via Gramsci 14, 43126 Parma, Italy; 2 Nuclear Medicine Unit, University Hospital of Parma, Via Gramsci 14, 43126 Parma, Italy

**Keywords:** Kidney transplantation, Vinflunine, Urothelial cancer, Immunosuppressive therapy, Pharmacological interaction

## Abstract

**Background:**

Patients undergone kidney transplantation present higher risk of Urothelial Carcinoma (UC) development and represent a subgroup of special interest. To date, vinflunine is the only drug approved in Europe for the treatment of advanced UC after failure of platinum-based chemotherapy. However, to our knowledge, no data on the concomitant administration of vinflunine and immunosuppressive agents are available.

**Case presentation:**

The patient, a 45 years old Caucasian male, presented poorly differentiated UC of the bladder recurred after initial cystectomy with abdominal lymphadenopathies evidenced by FDG-PET/CT. Previously, at the age of 22, he had post-glomerulonephritis renal failure and underwent kidney transplantation from deceased donor. Since then, he has been in treatment with immunosuppressive therapy. At the time of UC recurrence, he was on treatment with cyclosporine. After progression to platinum-based chemotherapy, he received second-line therapy with vinflunine resulting in a complete metabolic response after two cycles. However, despite several dose reductions, the patient experienced severe hematologic toxicity. The pharmacological interaction between vinflunine and cyclosporine, both metabolized by CYP 3A4, may explain the excellent result and the concomitant severe toxicity.

**Conclusions:**

Vinflunine is active on UC developed in kidney transplanted patients. However, special attention should be paid to concomitant administration with immunosuppressive agents that could result in increased toxicity.

## Background

In the USA, bladder cancer ranks 4^th^ and 11^th^ for cancer incidence in males and females, respectively. Whereas urothelial carcinoma (UC) accounts for 90 % of all bladder cancers, superficial UC, defined as non-muscle invasive cancer, represents 60 % of cases and requires local therapy with TURB (Trans-Urethral Resection of Bladder) and subsequent intravesical instillations [1, 2]. UC presents localized muscle invasion or distant metastases in 30 and 10 % of remaining cases, respectively. The management of muscle invasive disease includes neoadjuvant or adjuvant chemotherapy associated with cystectomy [2]. Despite surgery, 5-years survival varies between 36 and 48 %. In fact, peri-operative chemotherapy produce a benefit of approximately 5–7 % and a consistent part of patients develops recurrence of disease. The treatment of patients with recurrent or metastatic disease is currently represented by chemotherapy. As first-line therapy, Cisplatin and Gemcitabine (CG) demonstrated a similar efficacy of MVAC (Metotrexate, Vinblastine, Doxorubicin, Cisplatin) schedule with a more favourable toxicity profile [3]. Therefore, CG is the preferred option in first-line setting. However, considering that UC mainly develops in elderly patient (with a median age at diagnosis of approximately 70 years), the event of treating a patient un-fit for cisplatin is not unusual [4]. Un-fit patients are defined by the presence of at least one of the following: Eastern Cooperative Oncology Group Performance Status (ECOG PS) = 2, clearance of creatinine < 60 mL/min, grade ≥ 2 hearing loss or peripheral neuropathy [according to National Cancer Institute Common Terminology Criteria for Adverse Events (NCI-CTCAE)], heart failure (New York Heart Association functional classification class III). In these cases, carboplatin instead of cisplatin may be an option [5].

So far, vinflunine (VFL) is the only drug approved after failure of platinum-based treatment in Europe. It has been registered on the basis of a phase III trial that compared VFL versus best supportive care in second line setting, demonstrating a benefit of 2.6 months in median overall survival (OS) [6]. Principal VFL-related side effects include: constipation, anemia, neutropenia, vomiting and stomatitis. However, VFL has been proved to be acceptable also for elderly patients if dose reduction and granulocyte-colony stimulating factors (G-CSF) prophylaxis are observed [7]. With this evidence, the activity of a VFL based doublet as first-line therapy has been successfully explored in a phase II trial conducted in patients un-fit for cisplatin and a phase III trial comparing VFL-gemcitabine versus carboplatin-gemcitabine in the same setting is ongoing [8]. Even if preliminary data of check-point inhibition are encouraging also in UC patients, results from ongoing randomized trials will not be available before a couple of years [9, 10]. In conclusion, chemotherapy still play a crucial role in UC management and VFL is expected to be one of the protagonists in the next years scenario.

Smoking habit is the most important risk factor associated with the development of UC in west countries. Another well known risk factor is the exposition to aromatic amines and aniline derivatives [11]. Recently, a higher incidence of UC has been documented in a large series of patients who received renal transplantation [12]. Authors evidenced that patients diagnosed with bladder UC after renal transplantation were younger and presented more aggressive and advanced disease. The immune suppression required after transplantation is considered to support UC carcinogenesis process. Therefore, post-transplantation patients represent a particular subgroup of UC patients requiring special attention due to their comorbidity and concomitant immunosuppressive medications.

Here, we present a case report of a young kidney-transplanted patient who presented complete metabolic response after two cycles of second-line VFL chemotherapy. To our knowledge, this is the first report attesting the use of VFL in a patient under immunosuppressive therapy for kidney transplantation.

## Case presentation

At the time of diagnosis, the patient, a Caucasian male, was 45-years-old. His past medical history included hypertension, hyperuricemia, previous asymptomatic myocardial infarction and anti-HCV positivity. In 1988 post-glomerulonephritis renal failure occurred and he was treated with dialysis for 13 months. Subsequently, in 1989 the patient underwent kidney transplantation from deceased donor and since then immunosuppressive therapy was introduced. The oncological history started in February 2013, when a routinary abdominal ultra sound (US) exam, performed in another institution, evidenced a bladder wall thickening. Subsequent cystoscopy was performed showing suspect eteroplastic lesion. TURB revealed poorly differentiated transitional cell bladder carcinoma. In May 2013 TURB was repeated showing poorly differentiated carcinoma with neuroendocrine features. In June 2013, the patient had partial cystectomy plus lymphoadenectomy. The histology confirmed high grade UC with lymphatic invasion (stage pT2N0); immunohistochemistry resulted strongly positive for Ki-67 (100 %), negative for cromogranin-A, neuron-specific enolase, somatostatin receptor 2 antibody and synaptophysin. During follow-up, the patient underwent regular cystoscopies and in January 2014 the biopsy of a suspected mucosal area showed residual poorly differentiated carcinoma with focal CD56 stain. Therefore, in March 2014, radical cystectomy was completed with final histological report of poorly differentiated carcinoma with neuroendocrine features (pT1N0). During follow-up, a 18-F-fluorodeoxyglucose-positron emission tomography/computed tomography (FDG-PET/CT) performed in July 2014 showed the appearance of pathological uptake localized to confluent right iliac and retroaortic lymph nodes.

**Fig. 1:**
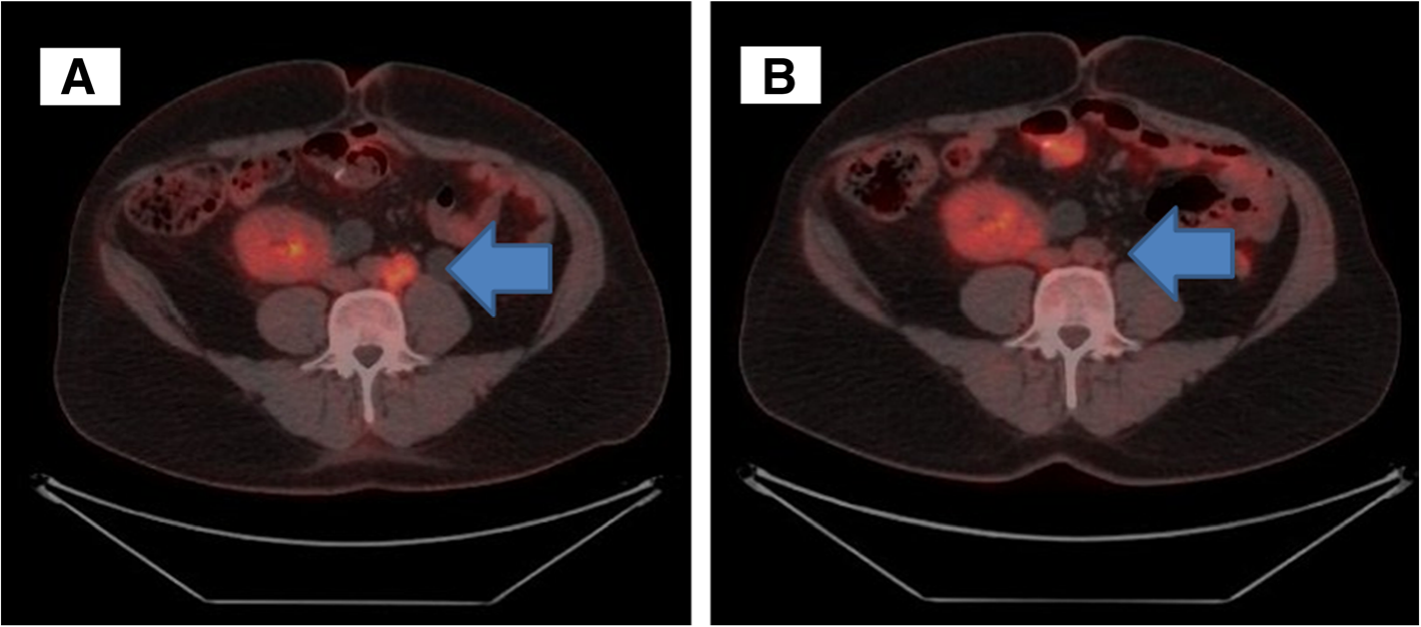
Para-aortic lymphodenopaties before therapy with vinflunine (VFL) (**a**) and after treatment (**b**)

**Fig. 2:**
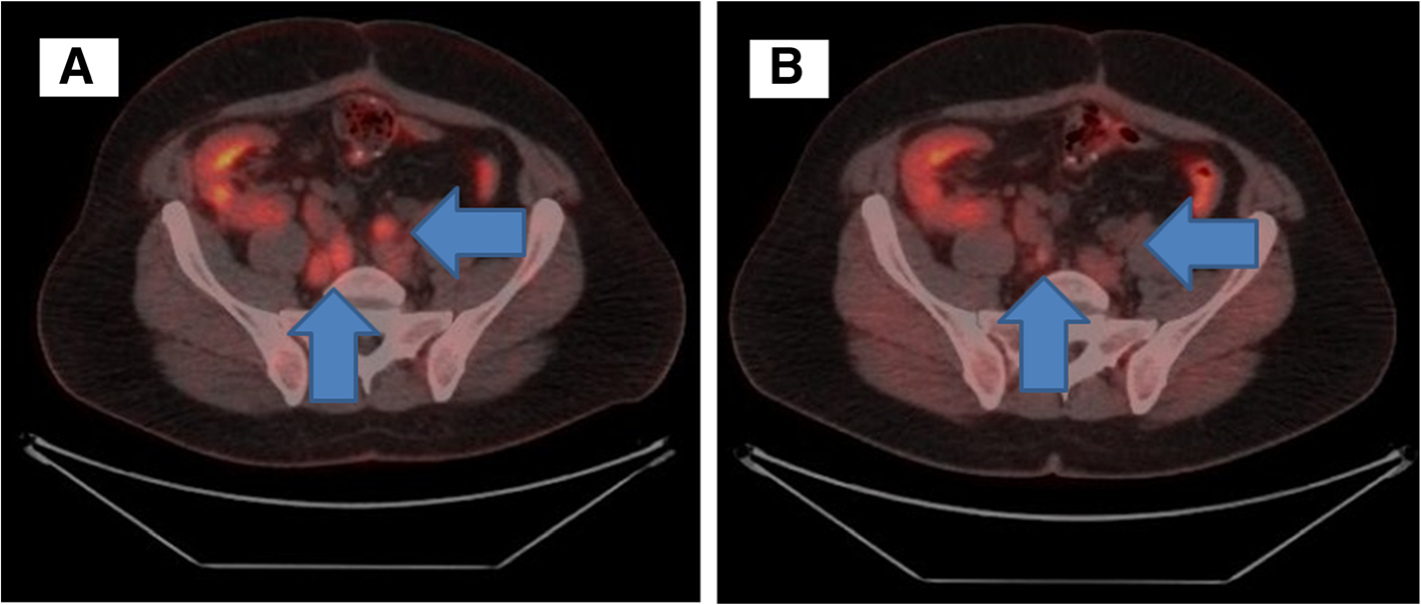
Bilateral common iliac lymphadenopathies before VFL therapy (**a**) and after treatment (**b**)

The patient came in our institution for the first time in August 2014 to discuss the initiation of first line chemotherapy. At that time, he was in therapy with cyclosporine 125 mg/die as immunosuppressive treatment. Therefore we considered to start chemotherapy with dose reduction as precaution. He started gemcitabine 1000 mg/m^2^ (day 1,8) on 27^th^ August 2014 and carboplatin AUC 3 (day 1) was added from the second cycle. During the treatment, the patient developed subsequent adverse events (AEs according to NCI-CTCAE version 4): anemia grade (G) 2, thrombocytopenia G1, neutropenia G3, ALT increased G2 and nausea G1. These AEs entailed dose delay and further dose reduction. A FDG-PET/CT was planned after 3 cycles and showed partial response. He completed 6 cycles of treatment but subsequent PET/CT, performed on February 2015, demonstrated progressive disease due to numeric increase of captating bilateral common iliac and paraortic adenopathies (Figs. 1a and 2a). On 27^th^ February 2015 the patient started second line treatment with VFL 280 mg/m^2^. After the first cycle the patient presented oral mucositis G1, loss of appetite and nausea G1 and prolonged G4 neutropenia that required precautionary hospital admission and administration of G-CSF and antibiotic prophylaxis. Considering the severe haematological toxicity, the second cycle was prescribed with further dose reduction (220 mg/m^2^) and G-CSF prophylaxis. Despite this, after the second cycle, neutropenia G4 and thrombocytopenia G4 occurred. In April 2015, FDG-PET/CT after two cycles documented complete metabolic response with disappearance of adenopathic pathological uptake (Figs. 1b and 2b). Chemotherapy proceeded with further dose reduction to 200 mg/m^2^ plus G-CSF prophylaxis and treatment was well tolerated with the exception of G2 temporary transaminases increase. In July 2015, before the 6^th^ cycle, patient presented gastroenteritis and diarrhea (seven stools/die for three consecutive days) with consequent acute renal failure (creatinine 5.5 mg/dL, clearance 23 ml/min). He was admitted to hospital and adequate hydration and supportive care was administered. After resolution of renal failure, which was considered not related to VFL, patient received the 6^th^ and last cycle. After treatment completion, FDG-PET/CT was repeated showing pathological captation of inter aortic-caval and right common iliac nodes. A multidisciplinary team decided for stereotactic radiotherapy on pathological nodes that the patient received in November 2015. A new FDG-PET/CT was planned for the end of January 2016, 2 months after the completion of radiotherapy, and revealed a partial metabolic response to radiotherapy. Then the patient was followed with close clinical and radiological controls.

## Conclusions

Here we report the case of a young kidney transplanted patient affected by advanced UC of the bladder and treated with second-line chemotherapy with VFL. To our knowledge, nothing has been previously reported regarding VFL therapy in patients with organ transplantation and immunosuppressive therapy. However, in patients undergone kidney transplantation evidence for an increased risk of aggressive UC development has recently been provided. Thus, the event of treating transplanted patient could be not so rare and sharing our experience could be relevant for other clinicians.

Accordingly to what reported by Yan and colleagues, the clinical behaviour of UC in our patient was highly aggressive [12]. This is consistent with the histo-pathological characteristics of the tumour such as neuroendocrine features, CD56 stain and extremely high proliferation index (Ki67 100 %). These factors were implicated in the two relapses occurred in our patient history that required radical cystectomy and the initiation of systemic chemotherapy later, when disease relapsed in advanced stage.

Our patient did not present any of the known principal negative prognostic factors for second line chemotherapy [13]. In fact, when chemotherapy was initiated for systemic recurrence Hb was higher than 10 g/dL, PS was 1, visceral and bone metastases were absent. As previously described, the patient experienced a significant response to VFL therapy considering the FDG-PET/CT after two cycles showed complete metabolic response. This was achieved despite the aggressiveness of histo-pathological features mentioned above and we argue that the absence of negative predictive factors could have play a role in reaching this result.

Nevertheless, treatment-related toxicity was the counterpart of the brilliant result in disease remission. In fact, during treatment patient presented serious side effects related to VFL that determined several dose reductions and required hospital admission and prophylaxis with antibiotic for severe neutropenia. Moreover, the patient also had an admission due to acute renal failure consequent to severe diarrhea complicating an episode of gastroenteritis. Although this event is not likely to be related to VFL therapy, it emphasizes the frailty of patients with kidney transplantation. Before starting therapy, we investigate the potential pharmacologic interaction between immunosuppressive therapy and VFL. During treatment with VFL, the patient was on therapy with cyclosporine as immunosuppressive agent. Both VFL and cyclosporine are prevalently metabolized by cytochrome CYP3A4 [14] (http://www.ema.europa.eu/docs/en_GB/document_library/EPAR_-_Product_Information/human/000983/WC500039604.pdf). Therefore, we hypothesize that during the concomitant administration, VFL and cyclosporine competed for the same cytochrome with a consequent reduction in their metabolism. For this reason, and considering the patient as frail, we decided to start therapy with 280 instead of 320 mg/m^2^. However, despite the initial dose reduction, our patient presented several serious AEs, suggesting us that drug interaction was more strong than expected. Our observation is limited by the fact that plasmatic levels of cyclosporine and of VFL have not been determined during treatment and therefore definitive conclusions can not be drawn. However, our hypothesis deserves to be evaluated in future patients.

In conclusion, patients with kidney transplantation present a higher risk of developing UC of the bladder. In this event, UC is highly aggressive and almost always spread with distant metastasis thus requiring a systemic chemotherapy treatment. Despite the young age, transplanted patients should be considered frail because of their past medical history and concomitant medications. VFL is the only chemotherapy registered after progression to platinum-based chemotherapy in UC. In this particular subset of patients, VFL is active and can be used in clinical practice. However, more attention should be paid to VFL dosage due to the high risk of toxicity probably related to pharmacological interaction between VFL and immunosuppressive concomitant therapy.

## Abbreviations

AE: Adverse Events
ALT: Alanine aminotransferase
AUC: Area Under Curve
CD56: Cluster of Differentiation 56
CG: Cisplatin and Gemcitabine
CYP3A4: Cytochrome P450 3A4
ECOG PS: Eastern Cooperative Oncology Group Performance Status
FDG-PET/CT: 18-F-Fluorodeoxyglucose-Positron Emission Tomography/Computed Tomography
G: Grade
G-CSF: Granulocyte-Colony Stimulating Factor
Hb: Haemoglobin
HCV: Hepatitis C Virus
MVAC: Metotrexate, Vinblastine, Doxorubicin, Cisplatin
NCI-CTCAE: National Cancer Institute Common Terminology Criteria for Adverse Events
TURB: Trans-Urethral Resection of Bladder
UC: Urothelial Carcinoma
US: Ultra Sound
VFL: Vinflunine
